# Differentially expressed and activated proteins associated with non small cell lung cancer tissues

**DOI:** 10.1186/s12931-015-0234-2

**Published:** 2015-06-24

**Authors:** E. Nigro, E. Imperlini, O. Scudiero, M.L. Monaco, R. Polito, G. Mazzarella, S. Orrù, A. Bianco, A. Daniele

**Affiliations:** CEINGE-Biotecnologie Avanzate Scarl, Via G. Salvatore 486, 80145 Naples, Italy; IRCCS SDN, Via E. Gianturco 113, 80142 Naples, Italy; Dipartimento di Medicina Molecolare e Biotecnologie Mediche, Università di Napoli Federico II, Via S. Pansini 5, 80131 Naples, Italy; Dipartimento di Scienze Cardio-Toraciche e Respiratorie, Seconda Università degli Studi di Napoli, Via L. Bianchi, 80131 Naples, Italy; Dipartimento di Scienze Motorie e del Benessere, Università di Napoli Parthenope, Via Amm. F. Acton 38, 80133 Naples, Italy; Cattedra di Malattie dell’Apparato Respiratorio, Dipartimento di Medicina e Scienze per la Salute “V Tiberio”, Università del Molise, Via De Sanctis, 86100 Campobasso, Italy; Dipartimento di Scienze e Tecnologie Ambientali Biologiche Farmaceutiche, Seconda Università degli Studi di Napoli, Via G. Vivaldi 42, 81100 Caserta, Italy; Present address: CEINGE-Biotecnologie Avanzate Scarl, Via G. Salvatore 486, 80145 Naples, Italy

**Keywords:** Lung cancer, Non small cell lung cancer [NSCLC], ERK1/2, AKT, IKBα, ΝF-κβ, Carbonic anhydrase I and II isoforms [CAI, II]

## Abstract

**Background:**

Lung cancer is a leading cause of mortality. The most common cancer subtype, non small cell lung cancer (NSCLC), accounts for 85-90 % all cases and is mainly caused by environmental and genetic factors. Mechanisms involved in lung carcinogenesis include deregulation of several kinases and molecular pathways affecting cell proliferation, apoptosis and differentiation. Despite advances in lung cancer detection, diagnosis and staging, survival rate still remains poor and novel biomarkers for both diagnosis and therapy need to be identified. In the present study, we have explored the potential of novel specific biomarkers in the diagnosis of NSCLC, and the over-expression/activation of several kinases involved in disease development and progression.

**Method:**

Lung tumor tissue specimens and adjacent cancer-free tissues from 8 NSCLC patients undergoing surgery were collected. The differential activation status of ERK1/2, AKT and IKBα/NF-κβ was analyzed. Subsequently, protein expression profile of NSCLC *vs* normal surrounding tissue was compared by a proteomic approach using LC-MS MS. Subsequently, MS/MS outputs were analyzed by the Protein Discoverer platform for label-free quantitation analysis. Finally, results were confirmed by western blotting analysis.

**Results:**

This study confirms the involvement of ERK1/2, AKT, IKBα and NF-κβ proteins in NSCLC demonstrating a significant over-activation of all tested proteins. Furthermore, we found significant differential expression of 20 proteins (R_sc_ ≥ 1.50 or ≤ −1.50) of which 7 are under-expressed and 13 over-expressed in NSCLC lung tissues. Finally, we validated, by western blotting, the two most under-expressed NSCLC tissue proteins, carbonic anhydrase I and II isoforms.

**Conclusion:**

Our data further support the possibility of developing both diagnostic tests and innovative targeted therapy in NSCLC. In addition to selective inhibitors of ERK1/2, AKT, IKBα and NF-κβ, as therapeutic options, our data, for the first time, indicates carbonic anhydrase I and II as attractive targets for development of diagnostic tools enabling selection of patients for a more specific therapy in NSCLC.

## Introduction

Lung cancer (LC) remains the leading cause of cancer death worldwide accounting for 14.1 million new cancer cases and 8.2 million deaths per year [1, 2]. Worldwide incidence appears to be variable but equally distributed with increasing trends among males and females [1, 2]. LC is stratified into two major subtypes, small cell lung cancer (SCLC) and non-small cell lung cancer (NSCLC), the latter representing 85-90 % of all cases of LC [3]. Response to current cytotoxic therapies has reached a plateau in terms of response rate and survival [4, 5]. The development of molecular profiling technologies to assess DNA, RNA, proteins and metabolites heralds a new era in the understanding of the molecular basis of non-small-cell lung carcinoma (NSCLC) leading to potential advances in management and treatment of lung cancer [6]. Novel molecular markers in non small-cell lung cancer (NSCLC) also include DNA damage repair genes. The pathogenesis of NSCLC cancer is complex and influenced by multiple factors including: a) environment (i.e. exposure to carcinogens, smoking habit, diet); b) genetic and epigenetic changes (such as p53 and EGFR gene mutations); c) pulmonary and systemic inflammatory conditions (such as concurrent Chronic Obstructive Pulmonary disease) [7–17]. At cellular level, activation status of several kinases involved in cell proliferation, apoptosis and inflammation is central to establishment and development of carcinogenesis. For example, constitutive activation of MEK-ERK, PI3K-AKT and/or PI3K-AKT-NF-κβ pathways plays a key role in oncogenesis and strongly promotes LC invasiveness [13–17]. AKT activation is present in 51 % of NSCLC although AKT mutations are rare (<1 %) [18, 19]. However, the role of MEK and PI3K pathways as prognostic and/or predictive markers for cancer remains controversial [20]. Today, the MEK and AKT inhibitors combined with chemotherapy are very promising for the treatment of several human cancers, including NSCLC [18, 19].

Despite the introduction of diagnostic tools such as computed tomography (CT) scans, Positrion Emission Tomography (PET) scan and bronchoscopy, the early diagnosis of LC remains unsatisfactory [20]. Circulating biochemical biomarkers such as carcinoembryonic antigen (CEA) TPA, CYFRA 21.1 etc. have a limited impact in early diagnosis whereas methylation-based assays may represent a more promising strategy for early detection and follow-up of NSCLC by means of a differential label-free proteomic analysis [21]. Actually, cancer detection, diagnosis and staging may be further improved by molecular selection [2, 21]. As current early detection procedures and treatments are unsatisfactory in terms of impact on quality of life and overall survival novel biomarkers need to be identified. In this context, the emerging “-omics” approach represents an important tool in the detection and quantitation of novel key-proteins as putative biomarkers for LC [3, 22].

In this study, we collected lung tumour specimens from 8 NSCLC chemotherapy naive patients undergoing surgery as well as surrounding cancer-free lung tissues and: a) investigated activation status of ERK, AKT, IKBα and ΝF-κβ; b) defined novel specific biomarkers for NSCLC diagnosis by means of a differential label-free proteomic analysis. We found: a) activation of all tested proteins and b) 20 proteins differentially expressed between NSCLC and controls. Finally, we validated, by western blotting, the two most differentially expressed proteins, carbonic anhydrase I and II (CAI, CAII) isoforms.

## Materials and methods

### Lung tissues sampling

NSCLC tumour tissue specimens and surrounding cancer-free tissue were collected from eight chemotherapy naïve patients who underwent thoracic surgery. Hystological analysis of tumour tissue specimens was conducted by pathologists at Monaldi Hospital of Naples and confirmed the diagnosis as follows: six adenocarcinoma and two adenosquamous carcinoma; surrounding cancer-free lung specimens showed evidence of airway remodeling and signs of mucociliary dysfunction and alveolar destruction consistent with diagnosis of Chronic Obstructive pulmonary diseases. Samples were immediately frozen at −80 °C. The study was approved by the local ethics committee and conducted in accordance with ethical principles stated in most recent version of the Declaration of Helsinki on the applicable guidelines for good clinical practices.

### Protein extraction

Lung tissues were homogenized in buffer containing 50 mM Tris–HCl, pH 7.5, 150 mM NaCl, 1 % [v/v] Triton X-100, 10 % [v/v] glycerol, 0.5 mM PMSF and complete mini protease inhibitor cocktail (Roche, Basel, Switzerland). Tissues were disrupted using a Dounce homogenizer and centrifuged at 16,000 g at 4 °C for 30 min. Protein concentrations were determined using Bradford’s reagent (Biorad Laboratories, CA, USA).

### Western blotting analysis

Protein extracts (30 μg) from each of 8 NSCLC and cancer-free lung tissue were incubated in Laemmli buffer with DTT, resolved on SDS-PAGE and then transferred onto nitrocellulose membranes (GE Healthcare, TX, USA) by Mini trans-blot electrophoresis transfer as previously described (Bio-Rad Laboratories, CA, USA) [23]. p-ERK1/2, ERK1/2, p-AMPK, AMPK, p-AKT, AKT, p-P38, P38, p-IKBα, IKBα antibodies were from Cell Signaling, Netherlands; NF-κβ antibody was from BD bioscience; CAI antibody was from Santa Cruz Biotechnology, MA, USA; CAII antibody was from Rockland, PA, USA; GAPDH and β-actin antibodies were from Sigma-Aldrich, MO, USA. Immunoblots were detected using the ECL-Advance Western Blotting Detection kit (GE Healthcare, TX, USA). Western blot images were scanned by PDquest 7.1 software (Bio-Rad Laboratories, CA, USA). Densitometric measurements were made with the Quantity One 4.5 tool (Bio-Rad Laboratories, CA, USA). Each experiment was performed at least three times in duplicate.

### In-gel digestion

Pooled protein extracts (100 μg) were obtained by mixing equal amounts of all 8 NSCLC samples as well as of corresponding controls. Both pooled protein samples were re-suspended in Laemmli buffer with 0.1 M DTT, incubated at 95 °C for 5 min and separated by SDS-PAGE. Molecular weight was estimated by using Precision Plus All Blue protein standards (Bio-Rad Laboratories, CA, USA). Protein electrophoretic patterns were then visualized using GelCode Blue Stain Reagent. Protein bands of interest were excised from gel lanes, crushed and washed first with acetonitrile (ACN) and then with 50 mM ammonium bicarbonate (AMBIC); enzymatic digestions were carried out as previously described [24]. In summary, protein samples were reduced in 10 mM DTT for 45 min at 56 °C and alkylated in 55 mM iodoacetamide in 50 mM AMBIC for 30 min at RT in the dark. Subsequently, gel particles were washed with 50 mM AMBIC and ACN and rehydrated in a modified trypsin solution (10 ng/μl) (Sigma, MO, USA) in 50 mM AMBIC pH 8.5, at 4 °C for 2 h. After removal of enzymatic solution, an aliquot of buffer solution was added and incubated at 37 °C for 18 h. The supernatant was collected, while gel pieces were subjected to further extraction in ACN at 37 °C for 15 min. Peptides obtained from extraction were pooled, vacuum-dried and resuspended in 0.2 % HCOOH before MS analysis.

### Mass spectrometry analysis

Peptide mixtures were analyzed by LC-MS MS using the LC/MSD Trap XCT Ultra (Agilent Technologies, CA, USA) equipped with a 1100 HPLC system and a chip cube (Agilent Technologies, CA, USA) as previously described [24]. Briefly, after loading, peptide mixture (8 μl in 0,2 % HCOOH) was pre-concentrated, washed at 4 μl/min in 40 nl enrichment column (Agilent Technologies chip) and separated on a RP-C18 column (75 μm × 43 mm) at a flow rate of 200 nl/min with a linear gradient of eluent B (2 % formic acid in acetonitryl) in eluent A (2 % formic acid) from 5 to 60 % in 60 min. Peptides were analyzed using data-dependent acquisition of MS scan (400–2000 m/z) followed by MS/MS scans of the three most abundant ions. Dynamic exclusion was used to acquire a more complete survey of the peptides. A permanent exclusion list of the most frequent peptide contaminants was included in the acquisition as previously described [25].

### Protein identification and quantitation

MASCOT software (Matrix Science, London, UK) was used for protein database searching as previously reported [26]. The searches were performed using the NCBI database and the following standard parameters: *Homo Sapiens*; one missed cleavage; carboxyamidomethylation of Cys, partial Met oxidation and putative modification of Gln to pyro-Glu, mass tolerance of 300 ppm on precursor ions, and 0.6 Da on the product ions. Individual ion scores >43 indicate identity or extensive homology (*p* < 0.05). For label-free quantitation, Mascot format text files were analyzed by Proteome Discoverer platform (version 1.3; Thermo Scientific, Bremen, Germany), interfaced with an in-house Mascot server (version 2.3, Matrix Science, London, UK). All peptides with FDR ≤ 0.01 and a peptide rank of 1 were included. Spectral counts (SpC) were used for estimating protein abundance and comparing the expression of the same protein between tumour and control tissues. SpC log Ratio (Rsc) and Normalized Spectral Abundance Factor (NSAF) were calculated as previously described [23].

### Statistical analysis

Data are expressed as means ± SD and median. The statistical significance was established at *p* <0.05. Two groups were compared with 2-tailed unpaired Student *t*-test.

## Results

### ERK1/2 and AKT kinases are significantly more activated in NSCLC tissues

**Fig. 1:**
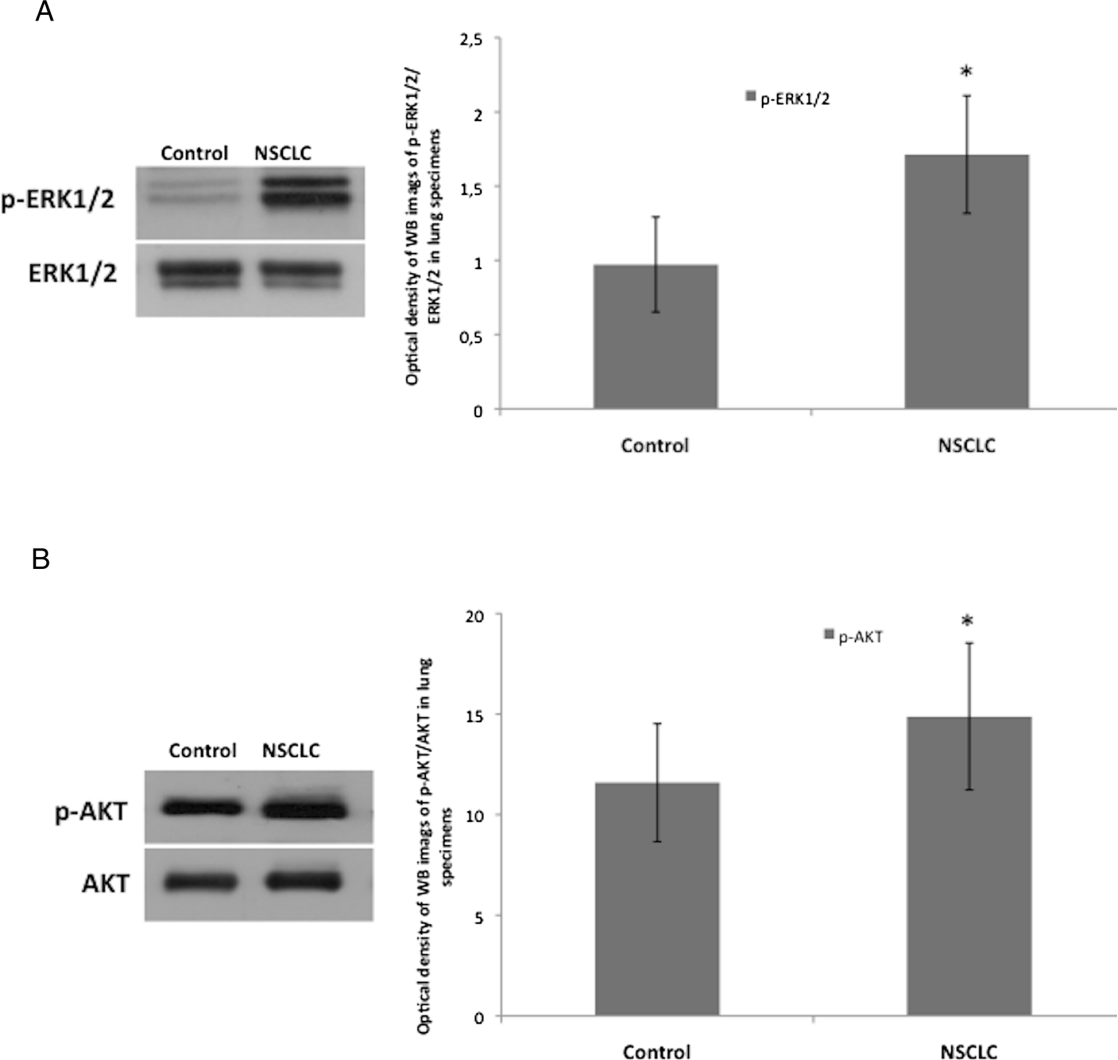
ERK1/2 and AKT kinases are significantly more activated in NSCLC than in control lung tissues. **a** One representative western blotting image and graphical representation of pixel quantization of p-ERK1/2 and relative total ERK1/2 of 8 lung tissue specimens. **b** One representative western blotting image and graphical representation of pixel quantization of p-AKT and relative total AKT of 8 lung tissue specimens. Each experiment was performed three times in duplicate.^*^ *= p* < 0.05 by *t*-test analysis. For other details see materials and methods

To analyze the expression profile of the most relevant proteins regulating cell survival proliferation and apoptosis in NSCLC, we evaluated the activation of MAPK, ERK1/2 and AKT kinases in LC tissue specimens. We demonstrated that p-ERK1/2 and p-AKT activation was statistically different between control cancer-free and NSCLC tissues; in fact, cancer specimens have a significantly higher expression of p-ERK1/2 and p-AKT compared to the control (*p* < 0.05) (Fig. 1a, and b, respectively). Ιn particular, p-ERK1/2 over-activation is 1,8 fold and p-AKT over-activation is 1,3-fold higher in NSCLC than in cancer-free tissues.

### NF-κβ and IKBa proteins are over expressed/activated in NSCLC tissues

**Fig. 2:**
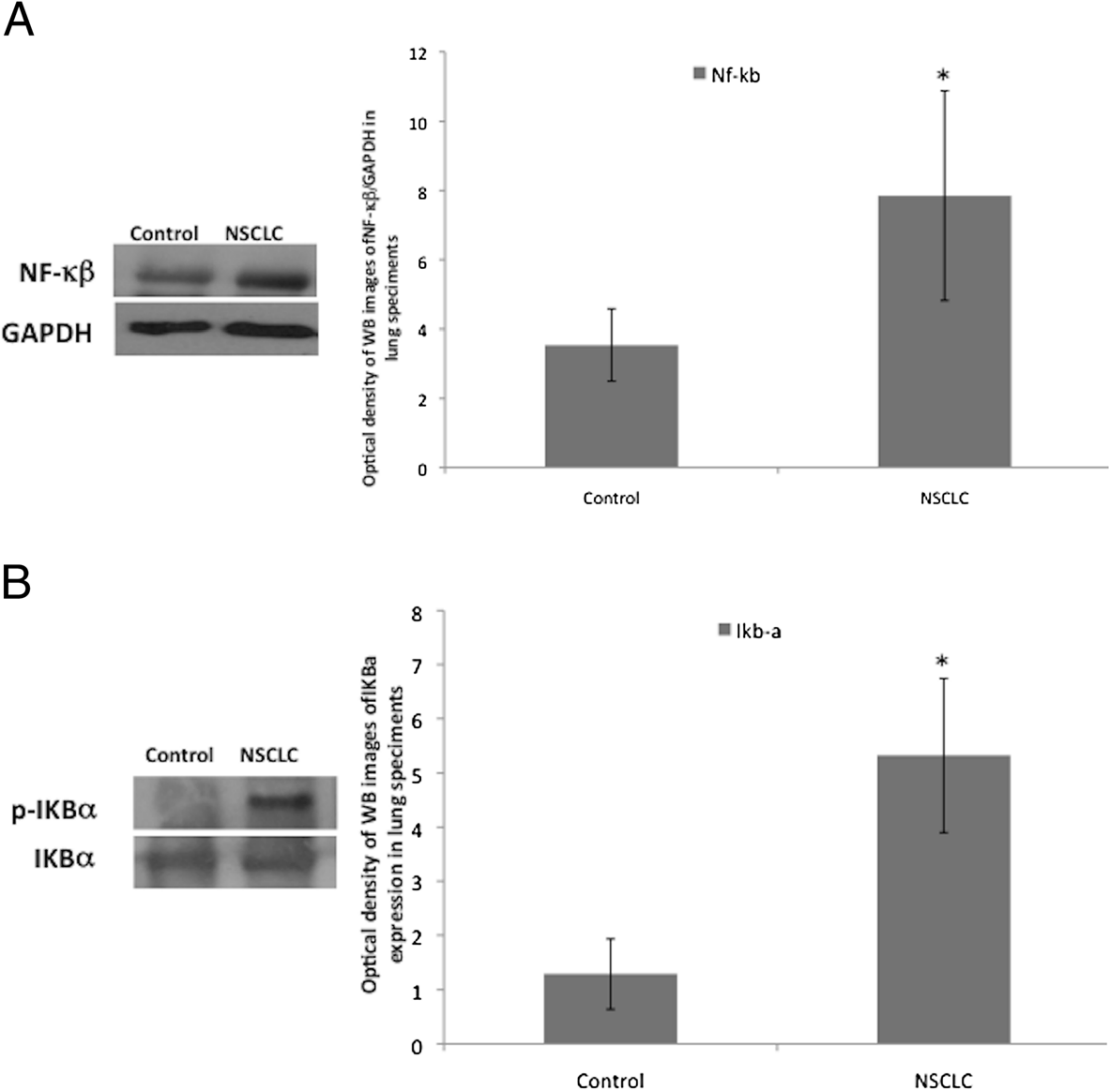
NF − κβ and p-IKBα are significantly more expressed in NSCLC than in control lung tissues. **a** One representative western blotting image and graphical representation of pixel quantization of NF − κβ and relative total GAPDH of 8 lung tissue specimens. **b** One representative western blotting image and graphical representation of pixel quantization of p-IKBα and total IKBα in 8 lung tissue specimens. Each experiment was performed three times in duplicate.^*^ *= p* < 0.05 by *t*-test analysis. For other details see materials and methods

We analyzed the expression of NF − κβ and p-IKBα, two proteins involved in the control of survival and inflammation and therefore potentially affected in cancer specimens. We found that NSCLC tissues have statistically higher expression of NF − κβ and IKBα compared to control cancer-free (Fig. 2a, b, respectively). Ιn particular, NF − κβ is over-expressed 2.2 fold while IKBα is 4-fold over-expressed in NSCLC respect to cancer-free tissues.

### Identification of differentially expressed proteins in NSCLC tissues

**Fig. 3:**
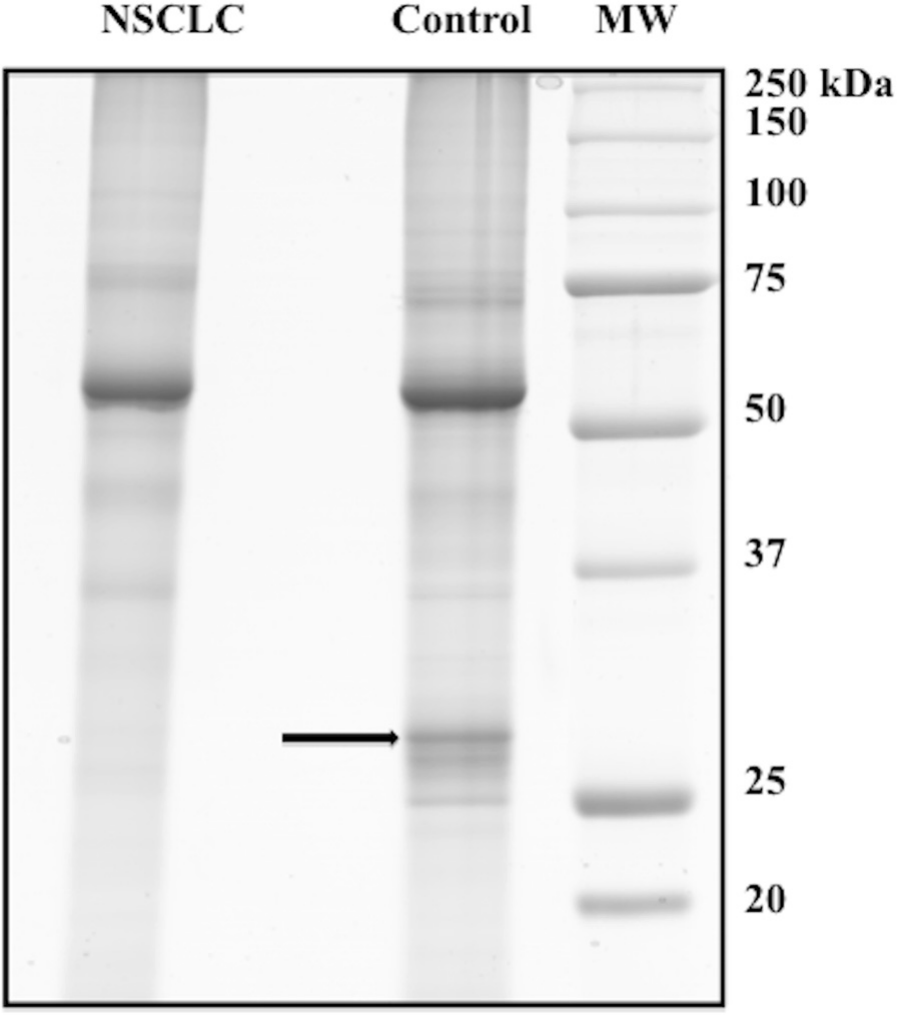
Differential protein expression profile between pooled tumour and control samples from 8 NSCLS patients (6 with histotype of AC and 2 with histotype of ASC). Arrow indicates protein band associated to CAI and CAII isoforms identification. For other details see materials and methods

We analyzed the protein expression profile of NSCLC and control cancer-free tissues in order to search for potentially LC biomarkers. Pooled protein extracts from 8 NSCLC and those from adjacent cancer-free lung tissues were fractionated onto a 10 % SDS-PAGE. As shown in Fig. 3, electrophoretic patterns between NSCLC and control extracts differed mainly in the low MW gel region. Protein bands from both lanes were excised individually, in-gel digested with trypsin, and analyzed by LC–MS/MS. We identified 38 proteins (see Table 1).

**Table 1:** Identified proteins in control and NSCLC tissues by MS analysis

Protein	Gene	Gene ID	MW^a^	Mascot Score^b^	Mascot Score^b^
				[Peptide]^c^	[Peptide]^c^
				NSCLC	Control
lactoferrin precursor	LTF	12083188	80214	946 [15]	298 [5]
transferrin	TF	37747855	79310	581 [9]	940 [15]
78 kDa glucose-regulated protein precursor	HSPA5	16507237	72402	507 [8]	503 [8]
moesin	MSN	4505257	67892	412 [8]	396 [7]
myeloperoxidase, isoform CRA_d	MPO	119614879	76923	331 [6]	73 [1]
protein disulfide-isomerase A4 precursor	PDIA4	4758304	73229	191 [3]	128 [2]
glyceraldehyde-3-phosphate dehydrogenase	GAPDH	31645	36201	581 [9]	375 [6]
annexin A2 isoform 2	ANXA2	18645167	38808	975 [16]	504 [10]
annexin A1	ANXA1	4502101	38918	587 [8]	522 [7]
L-lactate dehydrogenase A isoform 1	LDHA	5031857	36950	391 [7]	N. D.^d^
aldolase A	ALDOA	28614	39706	240 [4]	65 [1]
peroxiredoxin-1	PRDX1	4505591	22324	158 [3]	253 [5]
Aldo-keto reductase family 1 member C4	AKR1C4	308153631	37442	155 [3]	N. D.^d^
60S acidic ribosomal protein P0	RPLP0	4506667	34423	133 [2]	N. D.^d^
Actin, beta	ACTB	16359158	42078	103 [2]	238 [4]
alcohol dehydrogenase beta subunit	ADH1B	178098	40665	N. D.^d^	107 [2]
cathepsin D	CTSD	157879206	26511	151 [3]	131 [2]
stomatin	STOM	14715077	31958	112 [2]	N. D.^d^
apolipoprotein A-I, isoform CRA_b	APOA1	177827	23454	566 [9]	1057 [17]
ribosomal protein L7	RPL7	1335288	29907	170 [2]	75 [1]
tyrosine 3-monooxygenase/tryptophan 5-monooxygenase activation protein, gamma	YWHAG	380765197	28373	169 [2]	96 [1]
tyrosine 3-monooxygenase/tryptophan 5-monooxygenase activation protein, beta polypeptide variant	YWHAB	62089104	22563	48 [1]	113 [2]
triosephosphate isomerase 1	TPI1	136066	26894	633 [10]	394 [6]
proteasome subunit alpha type-6	PSMA6	8394076	27838	184 [3]	65 [1]
carbonic anhydrase 1	CA1	4502517	28909	104 [2]	482 [8]
carbonic anhydrase 2	CA2	15080386	29215	N. D.^d^	237 [4]
azurocidin	AZU1	28977	27093	130 [2]	N. D.^d^
phosphoglycerate mutase 1	PGAM1	4505753	28900	91 [2]	N. D.^d^
enoyl-CoA hydratase	EHHADH	1922287	31807	107 [2]	N. D.^d^
profilin-1	PFN1	4826898	15216	N. D.^d^	103 [2]
endoplasmic reticulum protein 29, isoform CRA_b	ERP29	119618398	29638	102 [2]	N. D.^d^
pre-serum amyloid P component	APCS	337758	25495	337 [5]	318 [5]
peroxiredoxin-6	PRDX6	134254698	25011	342 [6]	299 [5]
MHC class II antigen	HLA-DRB1	10185082	30525	N. D.^d^	183 [3]
MHC class I antigen	HLA-DQB	325656872	21947	N. D.^d^	150 [3]
Rab5c-like protein	RAB5C	508285	23781	101 [2]	97 [2]
proteasome subunit beta type-4	PSMB4	22538467	29242	N. D.^d^	211 [3]
rho GDP-dissociation inhibitor	ARHGDIB	56676393	23031	N. D.^d^	122 [2]

^a^Theoretical MW

^b^Protein score assigned by Mascot software is derived from ion scores that are -10*Log [p], where p is the probability that the observed match is a random event

^c^Number of peptides matched

^d^Not determined

In order to quantitatively compare the protein expression profiles of NSCLC and cancer-free lung tissues, MS/MS outputs were analyzed by the Protein Discoverer platform and submitted to label-free quantitation analysis. Table 2 contains the details of the label-free quantitation method based on spectral counting for protein abundance estimation. In addition to the Normalized Spectral Abundance Factor (NSAF) for each samples, it has been calculated the semi-quantitative parameter R_sc_, representing the log_2_ ratio between the protein expression level of control *vs* NSCLC lung tissues. This label-free procedure revealed 20 differentially expressed proteins with R_sc_ ≥ 1.50 or ≤ −1.50. In Table 2 such species are ranked from the highest R_sc_ value to the lowest. In particular, our analysis demonstrated that seven proteins with R_sc_ ≥ 1.50 are over-expressed in control, while 13 proteins R_sc_ ≤ −1.50 are over-expressed in NSCLC tissues.

**Table 2:** Spectral counting and protein ratios for differentially expressed proteins

Protein	Gene	NSAF^a^	NSAF^a^	R_sc_^b^
		NSCLC	Control	
carbonic anhydrase 2	CA2	0	0,00693	3,10
MHC class I antigen	HLA-DQB	0	0,0131	2,43
alcohol dehydrogenase beta subunit	ADH1B	0	0,00356	2,43
carbonic anhydrase 1	CA1	0,00559	0,0180	2,10
profilin-1	PFN1	0	0,00513	1,94
MHC class II antigen	HLA-DRB1	0	0,00931	1,94
rho GDP-dissociation inhibitor	ARHGDIB	0	0,00393	1,94
aldolase A	ALDOA	0,00591	0,000974	-1,61
ribosomal protein L7	RPL7	0,00869	0,00143	-1,61
enoyl-CoA hydratase	EHHADH	0,00250	0	-1,97
lactoferrin precursor	LTF	0,0124	0,00154	-2,25
proteasome subunit alpha type-6	PSMA6	0,0174	0,00144	-2,52
Aldo-keto reductase family 1 member C4	AKR1C4	0,00579	0	-2,71
stomatin	STOM	0,00604	0	-2,71
60S acidic ribosomal protein P0	RPLP0	0,00807	0	-3,20
phosphoglycerate mutase 1	PGAM1	0,0107	0	-3,20
endoplasmic reticulum protein 29, isoform CRA_b	ERP29	0,00894	0	-3,20
myeloperoxidase, isoform CRA_d	MPO	0,0184	0,00076	-3,48
L-lactate dehydrogenase A isoform 1	LDHA	0,00859	0	-3,57
azurocidin	AZU1	0,0161	0	-3,86

^a^Normalized Spectral Abundance Factor

^b^R_sc_ is calculated according to semi-quantitative parameter proposed by Old [42] and represents the log_2_ ratio between the protein expression level of control vs the protein expression level of NSCLC tissues. Proteins with R_sc_ ≥ 1,50 or ≤ -1,50 were considered differentially expressed

### Validation of carbonic anhydrase I and II isoforms

**Fig. 4:**
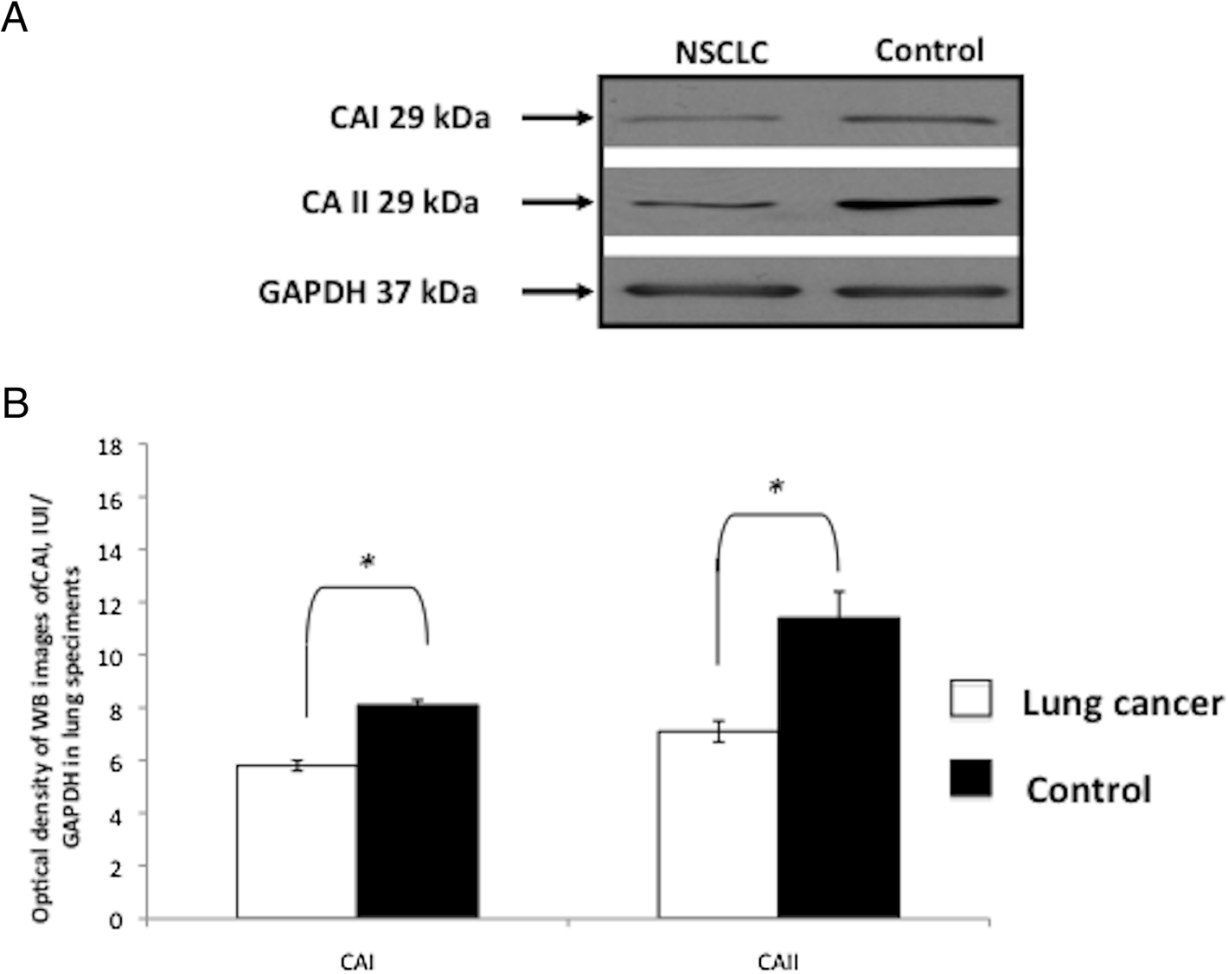
Western blot analysis confirms CAI and CAII as the two most differentially expressed proteins in NSCLC compared to control tissues. One representative western blot image (**a**) and graphical representation of pixel quantization (**b**) of CAI and CAII in lung tissues from 3 NSCLC patients (3 with histotype of AC and 1 with histotype of ASC). Each experiment was performed three times in duplicate^*^ *= p* < 0.05 by *t*-test analysis. For other details see materials and methods

Among proteins identified by MS analysis, we considered and validated carbonic anhydrase I (CAI) and II (CAII) isoforms. In fact, the two isoforms were identified in an intense protein band (MW between 25 kDa and 37 kDa), present in control and almost absent in the NSCLC (Fig. 3, as indicated by arrow); they were then quantified by label-free quantitation analysis. This procedure confirmed the strong over-expression of CAI and CAII (R_sc_ = 2,10 and R_sc_ = 3,10, respectively) in the control compared to NSCLC tissues (see Table 2). Western blotting analysis verified the significant under-expression of CAI and CAII proteins in NSCLC tissues compared to the control (Fig. 4a, b).

## Discussion

Surgical resection, when indicated, remains the best treatment option for LC patient whilst radiotherapy and chemotherapy, although effective, have plateaued in terms of response and survival [18, 19]. This highlights the necessity for earlier diagnosis and more specific therapies to be found. We focused on NSCLC, the most common LC subtype, investigating: a) the activation/expression status of some protein factors potentially involved in LC development, progression and therapy, and b) the differentially expressed lung proteins between NSCLC and cancer-free tissues in order to define novel biomarkers for NSCLC.

Our data confirmed that ERK1/2, AKT, IKBα and NF-κβ are proteins activated and/or over-expressed in NSCLC. RAS–ERK has, until now, been one of the most extensively studied signaling pathways as ERK1/2 pathway being often up‐regulated in different human tumors and therefore represents an attractive target for the development of anticancer drugs. Although the activation status of ERK1/2 has been largely studied in various cell lines, few previous reports have demonstrated an aberrant activation of ERK1/2 in human tumors especially in that of lung [27, 28]. In this study, we identified an over-phosphorylation of ERK1/2 in NSCLC confirming this kinase as a key molecular component implicated also in NSCLC cancer.

PI3K/AKT kinase pathway is another central regulator of cell metabolism, proliferation, and survival [29–33]. Furthermore, AKT is activated in pre-neoplastic and neoplastic lesions and has been linked to ineffectiveness of therapies resulting in poor prognosis [29, 30]. In particular, activation of PI3K/AKT pathway in NSCLC has been associated with increased cellular survival and resistance to chemotherapy and radiation, two important clinical problems encountered in several cancers [29]. Our data showed an over-activation of AKT in NSCLC cancer specimens. We analyzed not only AKT, but also the expression of IKBα and NF-kβ, other components connected to the PI3K/AKT pathway and involved in the control of survival and inflammation. The over-activation/expression of these molecules strongly supports that ERK1/2 and AKT pathways as well as IKBα/NF-κβ axis to be potential biomarkers for diagnosis/prognosis as well as development of novel targeted drugs in NSCLC. On the other hand, inactivation of NF-κβ, in combination with chemotherapeutic agents, leads to better therapeutic effects in several cancers [31, 32].

Our investigation also focused on the search for novel putative LC biomarkers. Proteomics in cancer research is a technology, by simultaneously examining thousands of proteins, can lead to the discovery of novel biomarkers for cancer [33]. We analyzed and compared the protein expression profile obtained from NSCLC and adjacent cancer-free lung tissues and, through a label-free proteomic approach, defined a set of 20 differentially expressed proteins: seven under-expressed and 13 over-expressed in NSCLC respect to control tissues. To our knowledge, some of them have been previously correlated to LC: aldolase A (ALDOA), phosphoglycerate mutase 1 (PGAM1), l-lactate dehydrogenase A isoform 1 (LDHA), enoyl-CoA hydratase (HADHA), and stomatin (STOM) [33–37]. Here we report the first experimental evidence of a significant increase of the above mentioned proteins in tissues from NSCLC patients. ALDOA, in fact, has been identified as a differentially expressed protein in the bronchoalveolar lavage of patients with LC and/or COPD [33]. Our data of PGAM1 over-expression agrees with that previously found in SCLC and adenocarcinomas (AC) [36]. In addition, LDHA over-expression supports recent findings from NSCLC mouse models both providing a translational impact to murine data and demonstrating the key role of LDHA in LC onset and progression [37]. Regarding the over-expression of HADHA protein, a significant positive correlation between HADHA expression and LC tumor was observed both in cisplatin-resistant LC cells and bioptic specimens from chemotherapy-resistant patients affected by NSCLC, or SCLC or AC has been reported [34]. As for STOM, our observation is in contrast with the only other report describing STOM role in carcinogenesis showing an under-expression of STOM protein n NSCLC tissues with positive lymph node metastasis [35].

The two most under-expressed proteins in NSCLC tissues, CAI and CAII, belong to a widespread family of 16 metallo-isoenzymes that catalyze the interconversion between carbon dioxide and the bicarbonate ion; these enzymes are involved in crucial physiological processes connected to respiration and transport of CO_2_/bicarbonate, in pH and CO_2_ homeostasis, and in many other metabolic reactions [38]. Furthermore, reduction of CAs levels causes CO_2_ intracellular retention with consequent increased acidification of extracellular pH, a typical condition observed in rapidly growing tumors [39, 40].

Recently, a differential expression of CA isoenzymes has been reported in some malignant tumors, and in particular CAIX has been shown to be prognostic indicator and a potentially important biomarker in the evaluation of cancer [39–41]. CAIX is constitutively up-regulated in several cancer types such as lung cancer, breast cancer etc. and therefore, IHC analysis of its expression represents a useful tool for cancer detection, diagnosis and staging in different tissues [41]. CAI and CAII are significantly less expressed in colorectal tumors, rectal carcinomas and pancreatic tumors, but over-expressed in nervous system tumors [40]. Recently, a critical role of CAII was reported on LC tumour growth, angiogenesis and survival [41]. To our knowledge, only one study has reported reduction of CAI and CAII expression in LC hypothesizing their role in tumour cell motility, tumour growth and metastasis formation [38]. Our data about CAI and CAII confirm and underline the role of these two isoforms in NSCLC and support a role for them for the first time as biomarkers for disease diagnosis and/or prognosis able to select patients for NSCLC therapy.

## Conclusions

Much effort is being made in the improvement of cancer detection, diagnosis and therapy by identifying more specific molecular biomarkers. In this context, we investigated and confirmed the involvement of ERK1/2, AKT, IKBα and NF-κβ proteins in NSCLC. Our data supports a role for these proteins as targets for development of novel drugs in LC treatment. Specifically, selective inhibitors of these molecular pathways could have important clinical implications for LC treatment. Comparing protein expression profile between NSCLC and adjacent cancer-free lung tissue, through a label-free proteomic approach, we found 20 differentially expressed proteins; among these we considered and validated CAI and CAII isoforms, the two most under-expressed proteins in cancer tissues. Our data strongly supports a role for these isoenzymes in the diagnosis and/or prognosis of NSCLC disease.

### Highlights

1. ERK1/2, AKT and IKBα/NF-κβ pathways are significantly over-activated in NSCLC tissues *vs* control [*p* < 0.05].
2. Proteomic analysis revealed 20 differently expressed proteins (7 under- and 13 over-expressed) between NSCLC and cancer-free lung tissues (≥1.50 R_sc_ ≤ −1.50).
3. Carbonic anhydrase I and II isoforms are strongly over-expressed in NSCLC tissues *vs* control (*p* < 0.05).
